# Tetra­ethyl­ammonium tricarbonyl­chlorido(quinoxaline-2-carboxyl­ato-κ^2^ *N* ^1^,*O*)rhenate(I)

**DOI:** 10.1107/S160053681002893X

**Published:** 2010-07-31

**Authors:** Janine Suthiram, Jan Rijn Zeevaart, Hendrik G. Visser, Andreas Roodt

**Affiliations:** a Radiochemistry, South African Nuclear Energy Corporation Ltd (Necsa), PO Box 582, Pretoria 0001, South Africa; b CARST, Northwest University, Mafikeng Campus, Private bag X2046, Mmabatho, 2735, South Africa; c Department of Chemistry, University of the Free State, PO Box 339, Bloemfontein 9300, South Africa

## Abstract

In the title compound, (C_8_H_20_N)[Re(C_9_H_5_N_2_O_2_)Cl(CO)_3_], the Re^I^ atom is coordinated facially by three carbonyl groups, the bidentate quinoxaline-2-carbaldehyde ligand and a chloride atom, forming a distorted octahedral geometry.. The crystal packing is controlled by C—H⋯O hydrogen bonding and π–π stacking inter­actions involving the benzene rings, with a centroid–centroid distance of 3.4220 (1) Å.

## Related literature

For synthetic background, see: Alberto *et al.* (1996 ▶). For related structures, see: Schutte *et al.* (2008 ▶); Wang *et al.* (2003 ▶); Alvarez *et al.* (2007 ▶); Brasey *et al.* (2004 ▶); Mundwiler *et al.* (2004 ▶); Feng *et al.* (2007 ▶); Suthiram *et al.* (2009 ▶). For bond-length data, see: Allen *et al.* (1987 ▶).
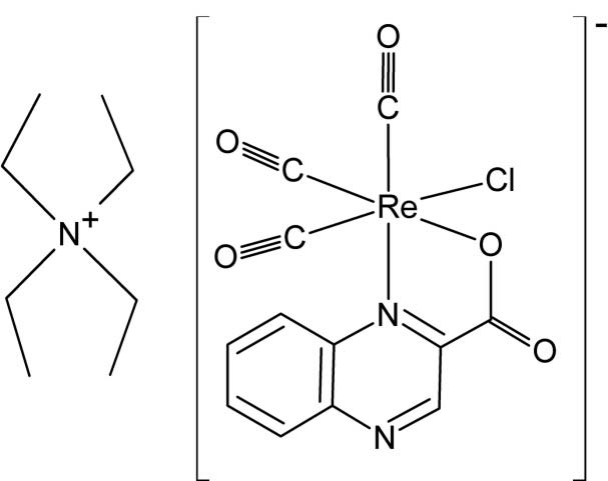


## Experimental

### 

#### Crystal data

(C_8_H_20_N)[Re(C_9_H_5_N_2_O_2_)Cl(CO)_3_]
*M* *_r_* = 609.08
Triclinic, 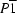

*a* = 8.402 (5) Å
*b* = 10.077 (5) Å
*c* = 13.495 (5) Å
α = 97.433 (5)°
β = 103.141 (5)°
γ = 90.686 (5)°
*V* = 1102.3 (9) Å^3^
*Z* = 2
Mo *K*α radiation
μ = 5.67 mm^−1^
*T* = 100 K
0.33 × 0.29 × 0.20 mm

#### Data collection

Bruker X8 APEXII 4K Kappa CCD diffractometer
Absorption correction: multi-scan (*SADABS*; Bruker, 2004 ▶) *T*_min_ = 0.169, *T*_max_ = 0.324
22501 measured reflections
5458 independent reflections
4988 reflections with *I* > 2σ(*I*)
*R*_int_ = 0.054

#### Refinement

*R*[*F* ^2^ > 2σ(*F* ^2^)] = 0.027
*wR*(*F* ^2^) = 0.065
*S* = 1.07
5458 reflections
275 parameters
12 restraints
H-atom parameters constrained
Δρ_max_ = 1.74 e Å^−3^
Δρ_min_ = −1.04 e Å^−3^

### 

Data collection: *APEX2* (Bruker, 2005 ▶); cell refinement: *SAINT-Plus* (Bruker, 2004 ▶); data reduction: *SAINT-Plus* and *XPREP* (Bruker, 2004 ▶); program(s) used to solve structure: *SIR97* (Altomare *et al.*, 1999 ▶); program(s) used to refine structure: *SHELXL97* (Sheldrick, 2008 ▶); molecular graphics: *DIAMOND* (Brandenburg & Putz, 2005 ▶); software used to prepare material for publication: *WinGX* (Farrugia, 1999 ▶).

## Supplementary Material

Crystal structure: contains datablocks global, I. DOI: 10.1107/S160053681002893X/pv2307sup1.cif

Structure factors: contains datablocks I. DOI: 10.1107/S160053681002893X/pv2307Isup2.hkl

Additional supplementary materials:  crystallographic information; 3D view; checkCIF report

## Figures and Tables

**Table 1:** Hydrogen-bond geometry (Å, °)

*D*—H⋯*A*	*D*—H	H⋯*A*	*D*⋯*A*	*D*—H⋯*A*
C10—H10⋯O5^i^	0.93	2.35	3.046 (5)	131

Symmetry code: (i) 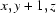.

**Fig. 1.**
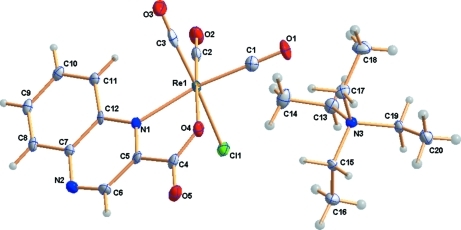
Molecular structure of the title compound. Displacement ellipsoids are drawn at the 50% probability displacement level.

**Fig. 2.**
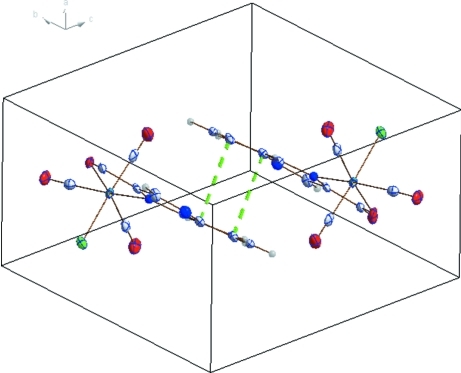
π-π -Stacking interactions of the title compound; cations have been omitted for clarity.

## supplementary crystallographic information

### Comment

The title complex, (C_8_H_20_N)[Re(C_9_H_5_N_2_O_2_)Cl(CO)_3_], forms a part of an ongoing investigation of the structural and kinetic behaviour of *fac*-Re(CO)_3_ compounds (Schutte *et al.*, (2008); Wang *et al.*, (2003); Alvarez *et al.*, (2007); Brasey *et al.*, (2004); Suthiram *et al.*, (2009)). It crystallized as an anionic Re^I^ compound and one tetraethylammonium counter ion in the asymmetric unit (Fig. 1). The Re—CO bond distances are well within the normal range (Allen *et al.*, 1987). The small bite angle O4—Re1—N1 of 74.71 (11) ° might be a reason for the distorted octahedral geometry around the metal centre. The crystal packing is controlled by C—H···O hydrogen bonding and π - π -stacking interactions involving the benzene rings, with a centroid-centroid distance of 3.4220 (1) Å (Fig. 2).

### Experimental

[NEt_4_]_2_[Re(CO)_3_Cl_3_] (150 mg, 0.235 mmol) was added to 30 ml me thanol to result in a suspension which was heated for a few minutes until the solution turned clear. Quinoxaline-2-carbaldehyde (41 mg, 0.235 mmol) was dissolved in 5 ml me thanol and slowly added to the reaction solution whilst stirring. K_2_CO_3_ (16.6 mg, 0.120 mmol) was added to the solution. The dark orange solution that formed was refluxed for 4 h after which the solvent was evaporated completely on a rotoevaporator. The resulting solid was redissolved in a minimum volume of dichloromethane, layered with diethyl ether and left to stand in a refrigerator. After several days red crystals suitable for X-ray diffraction were isolated. (Yield: 56 mg, 39%).

### Refinement

The methyl, methylene and aromatic H atoms were placed in geometrically idealized positions with C—H = 0.96, 0.97 and 0.93 Å, respectively and constrained to ride on their parent atoms, with *U*_iso_(H) = 1.5*U*_eq_(methyl C) and 1.2*U*_eq_(non-methyl C). The highest residual electron-density peak was located 0.85 Å from Re1 and the deepest hole was located 0.87 Å from Re1.

### Crystal data

**Table:**

(C_8_H_20_N)[Re(C_9_H_5_N_2_O_2_)Cl(CO)_3_]	*Z* = 2
*M**_r_* = 609.08	*F*(000) = 596
Triclinic, *P*1	*D*_x_ = 1.835 Mg m^−^^3^
Hall symbol: -P 1	Mo *K*α radiation, λ = 0.71069 Å
*a* = 8.402 (5) Å	Cell parameters from 9175 reflections
*b* = 10.077 (5) Å	θ = 2.7–28.3°
*c* = 13.495 (5) Å	µ = 5.67 mm^−^^1^
α = 97.433 (5)°	*T* = 100 K
β = 103.141 (5)°	Cuboid, red
γ = 90.686 (5)°	0.33 × 0.29 × 0.20 mm
*V* = 1102.3 (9) Å^3^	

### Data collection

**Table:**

Bruker X8 APEXII 4K Kappa CCD diffractometer	5458 independent reflections
Radiation source: sealed tube	4988 reflections with *I* > 2σ(*I*)
graphite	*R*_int_ = 0.054
φ & ω scans	θ_max_ = 28.3°, θ_min_ = 2.0°
Absorption correction: multi-scan (*SADABS*; Bruker, 2004)	*h* = −11→10
*T*_min_ = 0.169, *T*_max_ = 0.324	*k* = −13→13
22501 measured reflections	*l* = −17→17

### Refinement

**Table:**

Refinement on *F*^2^	12 restraints
Least-squares matrix: full	H-atom parameters constrained
*R*[*F*^2^ > 2σ(*F*^2^)] = 0.027	*w* = 1/[σ^2^(*F*_o_^2^) + (0.0211*P*)^2^ + 1.8438*P*] where *P* = (*F*_o_^2^ + 2*F*_c_^2^)/3
*wR*(*F*^2^) = 0.065	(Δ/σ)_max_ = 0.003
*S* = 1.07	Δρ_max_ = 1.74 e Å^−^^3^
5458 reflections	Δρ_min_ = −1.04 e Å^−^^3^
275 parameters	

### Special details

**Table:**

Geometry. All e.s.d.'s (except the e.s.d. in the dihedral angle between two l.s. planes) are estimated using the full covariance matrix. The cell e.s.d.'s are taken into account individually in the estimation of e.s.d.'s in distances, angles and torsion angles; correlations between e.s.d.'s in cell parameters are only used when they are defined by crystal symmetry. An approximate (isotropic) treatment of cell e.s.d.'s is used for estimating e.s.d.'s involving l.s. planes.

### Fractional atomic coordinates and isotropic or equivalent isotropic displacement parameters (Å^2^)

**Table:**

	*x*	*y*	*z*	*U*_iso_*/*U*_eq_	
Re1	0.528310 (18)	0.255408 (14)	0.783120 (11)	0.01364 (5)	
Cl1	0.81842 (12)	0.20551 (10)	0.85067 (7)	0.0205 (2)	
O3	0.1770 (4)	0.3314 (3)	0.6974 (2)	0.0304 (7)	
C3	0.3080 (5)	0.3001 (4)	0.7268 (3)	0.0208 (8)	
N3	0.0788 (4)	0.7963 (3)	0.8126 (2)	0.0139 (6)	
O1	0.4040 (4)	0.1531 (3)	0.9570 (2)	0.0279 (7)	
O4	0.5167 (3)	0.0596 (3)	0.6987 (2)	0.0182 (6)	
N1	0.6229 (4)	0.2820 (3)	0.6466 (2)	0.0124 (6)	
O2	0.5953 (4)	0.5310 (3)	0.9133 (2)	0.0290 (7)	
C2	0.5683 (5)	0.4271 (4)	0.8632 (3)	0.0191 (8)	
C13	−0.0041 (5)	0.8729 (4)	0.8902 (3)	0.0162 (8)	
H13A	0.0418	0.8469	0.9572	0.019*	
H13B	0.0218	0.9677	0.894	0.019*	
C15	0.0262 (5)	0.6478 (4)	0.7929 (3)	0.0198 (8)	
H15A	−0.091	0.6396	0.7647	0.024*	
H15B	0.0784	0.6037	0.7411	0.024*	
C1	0.4532 (5)	0.1938 (4)	0.8923 (3)	0.0195 (8)	
C20	0.0905 (5)	0.9922 (4)	0.7091 (3)	0.0236 (9)	
H20A	0.0551	1.0511	0.7609	0.035*	
H20B	0.0466	1.0193	0.643	0.035*	
H20C	0.2078	0.9963	0.7229	0.035*	
C14	−0.1876 (5)	0.8518 (4)	0.8673 (3)	0.0214 (8)	
H14A	−0.2342	0.8703	0.799	0.032*	
H14B	−0.2305	0.911	0.9156	0.032*	
H14C	−0.2148	0.7607	0.8729	0.032*	
C18	0.3657 (5)	0.7536 (5)	0.7873 (4)	0.0311 (10)	
H18A	0.3362	0.66	0.7679	0.047*	
H18B	0.4789	0.7645	0.8227	0.047*	
H18C	0.3486	0.7974	0.7269	0.047*	
C19	0.0298 (5)	0.8491 (4)	0.7098 (3)	0.0160 (8)	
H19A	−0.0886	0.8444	0.6882	0.019*	
H19B	0.071	0.7903	0.6594	0.019*	
C16	0.0657 (6)	0.5747 (4)	0.8857 (4)	0.0296 (10)	
H16A	0.1822	0.5716	0.909	0.044*	
H16B	0.0185	0.4852	0.8677	0.044*	
H16C	0.0215	0.621	0.9395	0.044*	
C17	0.2609 (5)	0.8150 (4)	0.8572 (3)	0.0207 (8)	
H17A	0.2872	0.7761	0.9207	0.025*	
H17B	0.2889	0.9102	0.874	0.025*	
O5	0.6377 (4)	−0.0666 (3)	0.5901 (2)	0.0236 (6)	
N2	0.7964 (4)	0.2711 (3)	0.4892 (3)	0.0185 (7)	
C12	0.6622 (4)	0.3988 (3)	0.6110 (3)	0.0116 (7)	
C10	0.6387 (5)	0.6348 (4)	0.6071 (3)	0.0155 (7)	
H10	0.5979	0.716	0.629	0.019*	
C11	0.6071 (4)	0.5229 (4)	0.6470 (3)	0.0141 (7)	
H11	0.5489	0.5285	0.6983	0.017*	
C5	0.6598 (5)	0.1677 (4)	0.5993 (3)	0.0143 (7)	
C6	0.7465 (5)	0.1632 (4)	0.5210 (3)	0.0184 (8)	
H6	0.7696	0.0799	0.4902	0.022*	
C7	0.7515 (4)	0.3910 (4)	0.5336 (3)	0.0139 (7)	
C4	0.6025 (5)	0.0410 (4)	0.6313 (3)	0.0167 (8)	
C8	0.7889 (5)	0.5102 (4)	0.4965 (3)	0.0159 (7)	
H8	0.8515	0.5073	0.4477	0.019*	
C9	0.7334 (4)	0.6286 (4)	0.5322 (3)	0.0163 (8)	
H9	0.7577	0.7064	0.5073	0.02*	

### Atomic displacement parameters (Å^2^)

**Table:**

	*U*^11^	*U*^22^	*U*^33^	*U*^12^	*U*^13^	*U*^23^
Re1	0.01848 (8)	0.01209 (8)	0.01132 (8)	−0.00015 (5)	0.00457 (6)	0.00329 (5)
Cl1	0.0230 (5)	0.0222 (5)	0.0152 (5)	0.0047 (4)	0.0008 (4)	0.0037 (4)
O3	0.0226 (16)	0.0426 (19)	0.0309 (18)	0.0056 (14)	0.0086 (14)	0.0175 (15)
C3	0.029 (2)	0.024 (2)	0.012 (2)	−0.0069 (17)	0.0097 (17)	0.0064 (16)
N3	0.0119 (15)	0.0165 (15)	0.0126 (16)	0.0013 (12)	0.0022 (12)	0.0002 (12)
O1	0.0362 (18)	0.0315 (17)	0.0191 (16)	−0.0072 (14)	0.0108 (13)	0.0076 (13)
O4	0.0267 (15)	0.0127 (12)	0.0158 (14)	−0.0025 (11)	0.0051 (12)	0.0044 (10)
N1	0.0110 (14)	0.0142 (14)	0.0113 (16)	−0.0001 (11)	0.0009 (12)	0.0025 (12)
O2	0.049 (2)	0.0187 (15)	0.0196 (16)	0.0003 (14)	0.0098 (14)	−0.0005 (12)
C2	0.025 (2)	0.023 (2)	0.0120 (19)	0.0013 (16)	0.0061 (16)	0.0083 (16)
C13	0.0191 (19)	0.0191 (18)	0.0112 (19)	0.0039 (15)	0.0058 (15)	0.0009 (14)
C15	0.022 (2)	0.0164 (18)	0.018 (2)	−0.0023 (15)	0.0019 (16)	−0.0025 (15)
C1	0.021 (2)	0.0199 (19)	0.017 (2)	−0.0021 (15)	0.0020 (16)	0.0022 (15)
C20	0.028 (2)	0.024 (2)	0.021 (2)	0.0015 (17)	0.0094 (18)	0.0061 (17)
C14	0.020 (2)	0.024 (2)	0.023 (2)	0.0041 (16)	0.0096 (17)	0.0043 (17)
C18	0.019 (2)	0.041 (3)	0.032 (3)	0.0088 (19)	0.0047 (19)	0.003 (2)
C19	0.0155 (18)	0.0215 (19)	0.0111 (19)	0.0023 (15)	0.0031 (14)	0.0023 (15)
C16	0.040 (3)	0.019 (2)	0.027 (2)	−0.0015 (18)	0.000 (2)	0.0060 (18)
C17	0.0113 (18)	0.028 (2)	0.019 (2)	−0.0025 (15)	−0.0029 (15)	0.0030 (17)
O5	0.0395 (18)	0.0115 (13)	0.0188 (15)	0.0045 (12)	0.0046 (13)	0.0015 (11)
N2	0.0205 (17)	0.0208 (16)	0.0154 (17)	0.0051 (13)	0.0069 (13)	0.0020 (13)
C12	0.0146 (17)	0.0127 (16)	0.0062 (17)	−0.0021 (13)	−0.0012 (13)	0.0028 (13)
C10	0.0173 (18)	0.0115 (16)	0.0144 (19)	−0.0003 (14)	−0.0027 (15)	0.0011 (14)
C11	0.0140 (17)	0.0175 (17)	0.0093 (18)	0.0012 (14)	−0.0004 (14)	0.0019 (14)
C5	0.0169 (18)	0.0162 (17)	0.0080 (18)	0.0031 (14)	−0.0016 (14)	0.0025 (14)
C6	0.023 (2)	0.0172 (18)	0.015 (2)	0.0064 (15)	0.0056 (16)	0.0002 (15)
C7	0.0134 (17)	0.0166 (17)	0.0106 (18)	0.0023 (14)	0.0003 (14)	0.0026 (14)
C4	0.022 (2)	0.0128 (17)	0.0136 (19)	0.0011 (14)	−0.0012 (15)	0.0040 (14)
C8	0.0151 (18)	0.0208 (18)	0.0115 (19)	−0.0007 (14)	−0.0005 (14)	0.0071 (15)
C9	0.0154 (18)	0.0160 (17)	0.016 (2)	−0.0038 (14)	−0.0023 (15)	0.0059 (14)

### Geometric parameters (Å, °)

**Table:**

Re1—C1	1.899 (4)	C14—H14C	0.96
Re1—C2	1.900 (4)	C18—C17	1.513 (6)
Re1—C3	1.917 (5)	C18—H18A	0.96
Re1—O4	2.136 (3)	C18—H18B	0.96
Re1—N1	2.211 (3)	C18—H18C	0.96
Re1—Cl1	2.4825 (16)	C19—H19A	0.97
O3—C3	1.145 (5)	C19—H19B	0.97
N3—C17	1.511 (5)	C16—H16A	0.96
N3—C19	1.521 (5)	C16—H16B	0.96
N3—C13	1.521 (5)	C16—H16C	0.96
N3—C15	1.527 (5)	C17—H17A	0.97
O1—C1	1.164 (5)	C17—H17B	0.97
O4—C4	1.281 (5)	O5—C4	1.225 (4)
N1—C5	1.319 (5)	N2—C6	1.315 (5)
N1—C12	1.390 (4)	N2—C7	1.374 (5)
O2—C2	1.160 (5)	C12—C11	1.405 (5)
C13—C14	1.508 (5)	C12—C7	1.413 (5)
C13—H13A	0.97	C10—C11	1.360 (5)
C13—H13B	0.97	C10—C9	1.417 (5)
C15—C16	1.510 (6)	C10—H10	0.93
C15—H15A	0.97	C11—H11	0.93
C15—H15B	0.97	C5—C6	1.410 (5)
C20—C19	1.526 (5)	C5—C4	1.506 (5)
C20—H20A	0.96	C6—H6	0.93
C20—H20B	0.96	C7—C8	1.417 (5)
C20—H20C	0.96	C8—C9	1.356 (5)
C14—H14A	0.96	C8—H8	0.93
C14—H14B	0.96	C9—H9	0.93
			
C1—Re1—C2	88.06 (17)	C17—C18—H18A	109.5
C1—Re1—C3	88.08 (17)	C17—C18—H18B	109.5
C2—Re1—C3	90.12 (17)	H18A—C18—H18B	109.5
C1—Re1—O4	93.58 (14)	C17—C18—H18C	109.5
C2—Re1—O4	172.60 (14)	H18A—C18—H18C	109.5
C3—Re1—O4	97.15 (15)	H18B—C18—H18C	109.5
C1—Re1—N1	167.94 (14)	N3—C19—C20	115.4 (3)
C2—Re1—N1	103.18 (14)	N3—C19—H19A	108.4
C3—Re1—N1	96.14 (14)	C20—C19—H19A	108.4
O4—Re1—N1	74.71 (11)	N3—C19—H19B	108.4
C1—Re1—Cl1	94.68 (13)	C20—C19—H19B	108.4
C2—Re1—Cl1	88.98 (13)	H19A—C19—H19B	107.5
C3—Re1—Cl1	177.06 (11)	C15—C16—H16A	109.5
O4—Re1—Cl1	83.70 (8)	C15—C16—H16B	109.5
N1—Re1—Cl1	81.35 (8)	H16A—C16—H16B	109.5
O3—C3—Re1	176.6 (4)	C15—C16—H16C	109.5
C17—N3—C19	111.5 (3)	H16A—C16—H16C	109.5
C17—N3—C13	106.6 (3)	H16B—C16—H16C	109.5
C19—N3—C13	110.3 (3)	N3—C17—C18	114.5 (3)
C17—N3—C15	110.7 (3)	N3—C17—H17A	108.6
C19—N3—C15	106.6 (3)	C18—C17—H17A	108.6
C13—N3—C15	111.2 (3)	N3—C17—H17B	108.6
C4—O4—Re1	117.4 (2)	C18—C17—H17B	108.6
C5—N1—C12	117.2 (3)	H17A—C17—H17B	107.6
C5—N1—Re1	112.5 (2)	C6—N2—C7	115.9 (3)
C12—N1—Re1	129.9 (2)	N1—C12—C11	120.9 (3)
O2—C2—Re1	178.7 (4)	N1—C12—C7	119.3 (3)
C14—C13—N3	115.2 (3)	C11—C12—C7	119.7 (3)
C14—C13—H13A	108.5	C11—C10—C9	120.4 (3)
N3—C13—H13A	108.5	C11—C10—H10	119.8
C14—C13—H13B	108.5	C9—C10—H10	119.8
N3—C13—H13B	108.5	C10—C11—C12	120.1 (4)
H13A—C13—H13B	107.5	C10—C11—H11	120
C16—C15—N3	115.3 (3)	C12—C11—H11	120
C16—C15—H15A	108.4	N1—C5—C6	121.8 (3)
N3—C15—H15A	108.4	N1—C5—C4	117.0 (3)
C16—C15—H15B	108.4	C6—C5—C4	121.1 (3)
N3—C15—H15B	108.4	N2—C6—C5	123.3 (3)
H15A—C15—H15B	107.5	N2—C6—H6	118.4
O1—C1—Re1	177.9 (4)	C5—C6—H6	118.4
C19—C20—H20A	109.5	N2—C7—C12	122.2 (3)
C19—C20—H20B	109.5	N2—C7—C8	118.7 (3)
H20A—C20—H20B	109.5	C12—C7—C8	119.0 (3)
C19—C20—H20C	109.5	O5—C4—O4	127.0 (4)
H20A—C20—H20C	109.5	O5—C4—C5	118.5 (4)
H20B—C20—H20C	109.5	O4—C4—C5	114.5 (3)
C13—C14—H14A	109.5	C9—C8—C7	120.1 (4)
C13—C14—H14B	109.5	C9—C8—H8	120
H14A—C14—H14B	109.5	C7—C8—H8	120
C13—C14—H14C	109.5	C8—C9—C10	120.6 (3)
H14A—C14—H14C	109.5	C8—C9—H9	119.7
H14B—C14—H14C	109.5	C10—C9—H9	119.7
			
C1—Re1—O4—C4	−159.2 (3)	C15—N3—C19—C20	−172.6 (3)
C3—Re1—O4—C4	112.3 (3)	C19—N3—C17—C18	−55.5 (4)
Cl1—Re1—O4—C4	−64.9 (3)	C13—N3—C17—C18	−176.0 (3)
C2—Re1—N1—C5	157.7 (3)	C15—N3—C17—C18	63.0 (4)
C3—Re1—N1—C5	−110.7 (3)	C5—N1—C12—C11	171.0 (3)
Cl1—Re1—N1—C5	70.8 (2)	Re1—N1—C12—C7	167.3 (2)
C1—Re1—N1—C12	−174.0 (6)	N1—C12—C11—C10	−176.3 (3)
C3—Re1—N1—C12	76.0 (3)	Re1—N1—C5—C6	−169.4 (3)
O4—Re1—N1—C12	171.8 (3)	C12—N1—C5—C4	−174.2 (3)
Cl1—Re1—N1—C12	−102.4 (3)	Re1—N1—C5—C4	11.5 (4)
C17—N3—C13—C14	−172.0 (3)	C4—C5—C6—N2	178.8 (4)
C19—N3—C13—C14	66.8 (4)	C6—N2—C7—C8	−174.6 (3)
C15—N3—C13—C14	−51.2 (4)	C11—C12—C7—N2	−174.3 (3)
C17—N3—C15—C16	57.8 (5)	N1—C12—C7—C8	179.0 (3)
C19—N3—C15—C16	179.3 (3)	Re1—O4—C4—O5	164.6 (3)
C13—N3—C15—C16	−60.5 (4)	N1—C5—C4—O5	−178.6 (3)
C17—N3—C19—C20	−51.7 (4)	C6—C5—C4—O4	−175.9 (3)
C13—N3—C19—C20	66.6 (4)	N2—C7—C8—C9	174.1 (3)

### Hydrogen-bond geometry (Å, °)

**Table:**

*D*—H···*A*	*D*—H	H···*A*	*D*···*A*	*D*—H···*A*
C10—H10···O5^i^	0.93	2.35	3.046 (5)	131

Symmetry codes: (i) *x*, *y*+1, *z*.
